# Still Left Behind: How Behavior Analysts Can Improve Children’s Access, Equity, and Inclusion to Their Entitled Education

**DOI:** 10.1007/s40617-024-00992-4

**Published:** 2024-10-10

**Authors:** Bradley Stevenson, Keri Bethune, Rita Gardner

**Affiliations:** 1 Melmark Carolinas, Charlotte, NC USA; 2 Melmark, Berwin, PA USA

**Keywords:** Advocacy, Behavior analysis, School exclusion, Individuals with Disabilities Education Act, Special education, Discipline, Diversity, Access, Equity, Inclusion

## Abstract

In 1968, a group of professionals commissioned a task force to study the issue of children being excluded from school in the city of Boston, MA (Task Force on Children Out of School, 1970). What they found shocked them: thousands of children were systematically excluded from attending school or accessing meaningful instruction based on cultural, physical, and mental and behavioral differences. However, despite the advancement of legal protections and improved methods to educate even the most complex students, many coming from behavior analysis, children across the country still face school exclusion for essentially the same reasons. Coordinated advocacy is needed urgently to address this issue. This article discusses the history of school exclusion, the advancements that should allow us to prevent it, and a description of advocacy efforts behavior analysts should engage in to prevent school exclusion from occurring.

In 1968, there was a crisis among children in Boston. It was becoming evident to a group of social workers, mental health professionals, and other community leaders that certain children were being denied access to their education. The problem was so widespread, and the effects of school exclusion on children so severe, this group declared, “The problem of exclusion from, and within, the school system constitutes an emergency situation” (Task Force on Children Out of School, 1970, p. iii). As such, representatives from various stakeholders came together to address the issue. They commissioned a Task Force on Children Out of School (1970) to study the issue in greater depth.

The Task Force did not reserve their criticism solely for the schools, stating that although the systematic exclusion originated within the school, the community also bore responsibility for failing to help the children. Other services, such as mental health clinics, could not support these children quickly or help them access their education (Task Force on Children Out of School, 1970). Even more than that, the task force found the community at large was complicit, writing that, “While our findings are an indictment of the system of public education, above all else they are an indictment of the total community whose indifference and inaction promotes this shameful condition” (p. iii). They concluded that school exclusion was not only a school problem but also a community problem, arguing, “The burden falls upon us all to mobilize to meet the needs of our children” (p. 71).

## The Movement to Prevent School Exclusion

Mobilize they did. Four years after that initial meeting in 1972, the Massachusetts legislature passed Chapter 766, the first special education law in the country, guaranteeing *all* children the right to education to the “maximum possible development” (Massachusetts Teachers Association, 2008). The legal protections within that law were central to the efforts toward curbing school exclusion, not just in the city of Boston, but across the Commonwealth of Massachusetts.

Given that the exclusion of children with disabilities from school was not unique to the state of Massachusetts, neither was the movement to prevent it. In fact, in 1972, the U.S. Eastern District of Pennsylvania decided the case of *Pennsylvania Association for Retarded Citizens (PARC) *v.* Commonwealth of Pennsylvania* (hereafter *PARC *v.* Pennsylvania*; Ross, 2022). PARC brought the case against the state of Pennsylvania on behalf of 14 families with children with intellectual disabilities who could not access public schools based on their child’s disability. PARC challenged the state laws that permitted schools to deny education to children who do not reach the mental age of five, or the average intelligence of people aged five, by the time they begin first grade. Both sides settled following the testimony of expert witnesses on PARC’s behalf, and the U.S. District Court approved the consent decree. Thereafter, it was decided the state could not deny an individual’s right to equal access to education based on intellectual or developmental disability status.

*PARC* v. *Pennsylvania* was one of the first cases to establish that people born with an intellectual disability should have the same access to education as the rest of the population but not the last. Twenty-seven federal lawsuits from across the country involving disabilities and education followed *PARC *v.* Pennsylvania,* leading to Congress passing legislation to mandate that all states grant equal access to education for students with disabilities (Ross, 2022). As a result of this movement towards ensuring education access, in 1975, the federal government passed what would become the Individuals with Disabilities Education Act (2004; IDEA), incorporating many of the same protections from both the Massachusetts Chapter 766 special education law and various lawsuits from across the country into federal law making them applicable to every child in the country.

Over the years, progress has continued to expand protections for children with disabilities to ensure access to a free appropriate public education (FAPE) in the least restrictive environment (LRE) across the country. Since the passing of the initial federal legislation, the U.S. Congress has added additional areas of eligibility, expanded the ages during which children are eligible for services, and required manifestation determination reviews for children with disabilities who exhibit challenging behaviors, all of which have improved ongoing access to special education (Office of Special Education Programs, 2020). Guidance has also been issued to prevent school exclusion, such as the recent document clarifying that children facing informal removals from schools are entitled to the same protections as those facing formal disciplinary exclusion (Office of Special Education Programs, 2022). In addition, the judicial system has joined the movement to broaden protections for children with disabilities to ensure access to their education. The most recent high-profile example of this is the *Endrew F. *v.* Douglas County School District* (2017) decision. In that case, the Supreme Court rejected the school district's contention that an education that conferred *de minimis* benefit was appropriate. The Court stated that each child should be able to access an education with goals that are appropriately ambitious in light of their unique circumstances. They even cited the issue of school exclusion in their rationale, stating that requiring meaningful progress, “reflects the broad purpose of the IDEA, an ‘ambitious’ piece of legislation enacted ‘in response to Congress’ perception that a majority of handicapped children in the United States ‘were either totally excluded from schools or [were] sitting idly in regular classrooms awaiting the time when they were old enough to ‘drop out’” (*Endrew F.* v. *Douglas County School District*, 2017).

As special education law was developing, so was the field of applied behavior analysis (ABA), creating our technology to educate even the most complex learners. As ABA became a new field studying behavior and learning, a natural pairing arose between behavior analysis and the field of education. In fact, four of the eight research studies in the first issue of the *Journal of Applied Behavior Analysis* (*JABA*) occurred in classrooms (Wolf, 1968).

As the field of ABA grew, behavior analysis developed a rich history of research in education settings. As early as 1965, Ogden Lindsey left Harvard Medical School as a psychologist to move to the University of Kansas to work in teacher training and went on to publish multiple works on Precision Teaching and other effective teaching practices. Many well-regarded and published leaders in the field of ABA work or worked in education. These include George Sugai, Rob Horner, John Cooper, Timothy Heron, William Heward, and Nancy Neef. Moreover, many behavior analytic graduate programs are housed within education departments such as the University of Georgia, Endicott College, Vanderbilt University, and the Ohio State University. Multiple textbooks and manuscripts have focused on integrating behavior-analytic methods in education settings. Finally, multiple studies have found many evidence-based practices in education are behavior analytic. These include, but are not limited to, functional behavior assessment, pivotal response training, reinforcement, functional communication training, antecedent strategies, behavioral packages, systematic instruction utilizing prompting and fading, task analysis, and discrete trial training (Hume et al., 2021).

Last, it is widely acknowledged that behavior challenges that pose a barrier to children accessing FAPE must be addressed. Due to the field’s rich tradition of successfully identifying controlling variables for and drafting interventions to address challenging behavior, these behavior analytic methods were incorporated into federal law. This can be seen in the IDEA (2004) specifically using the term “functional behavior assessment” to describe required supports for students with disabilities who exhibit challenging behavior that interferes with their learning or the learning of others.

## The Crisis of School Exclusion Persists

Despite this progress to include children in school, a shocking number of children with disabilities are still being excluded from school in a variety of ways. This includes using “off-the-books” suspensions, modifying students’ days by reducing the hours they can attend school, and placing children in a homebound setting for behavioral purposes (Office of Special Education & Rehabilitative Services, 2022). First, Meredith Kolodner and Annie Ma (2022) highlight the problem of “off-the-book” suspensions, or informal removals, routinely occurring in schools across the county. This is a process where educators exclude children from school, often due to challenging behaviors, by simply asking caregivers to pick them up early, drop them off late, or otherwise remove them from classrooms without officially recording it as a suspension. Through interviews with 20 families across 10 states, the reporters chronicled how these informal removals occur and affect the families. In that article, Ginny Fogg, an attorney at Disability Rights North Carolina, describes the problem of informal removals as “pervasive,” stating,

The reason for that is that most parents don’t know their rights and the consequence for the school system is not enough to make them not do it . . . the “remedy isn’t, ‘You just can’t go to school. . . .’ The law was enacted 50 years ago to prevent this very outcome—that students with disabilities aren’t allowed to go to school and participate in an education. (Kolodner & Ma, 2022).

Even when educators and administrators use official disciplinary channels, students with disabilities can still be excluded from school through modified day practices. A modified school day is considered a “partial homebound” placement where the student is not allowed to remain in school for an entire school day, and the school day is officially modified on their individualized education program (IEP). The case of Lewiston Public Schools in Maine illustrates how pervasive this practice can be. In 2016, Disability Rights Maine filed a complaint to the U.S. Department of Justice about using modified day placements in Lewiston. After investigation, the Department of Justice found, “the district routinely shortened the school day for students with disabilities without considering their individual needs or exploring supports to keep them in school for the full day” (AP News, 2021). They continued to describe the practice of excluding children from school due to challenging behaviors that were manifestations of their disability as “systemic and discriminatory” (AP News, 2021).

Another recent lawsuit filed in Oregon found similar practices. That lawsuit, which was settled, documented how Oregon illegally denied hundreds of students with disabilities the right to a full school day. It highlights a series of specific cases where students’ days were shortened due to challenging behaviors. The suit goes further to describe the problem as widespread and argues the Oregon Department of Education was aware of the problem and did not take steps to remedy the issue (Stites, 2022).

Finally, another tactic that excludes students with disabilities from education is to place them on “homebound.” Homebound placements have been increasingly used for students with disabilities who display challenging behavior in school. A student on “homebound” is not permitted to attend school, but instead receives instruction from a teacher for a small portion of time, often as little as an hour a week, in their home or a hospital setting. A recent study by Disability Rights North Carolina and the North Carolina Department of Public Instruction found that over 800 students were placed on homebound in North Carolina alone during the 2016 school year, often receiving as little as one to three hours of instruction per week (Disability Rights North Carolina & North Carolina Department of Public Instructions, 2017). At the same time, another 300 had their days officially shortened.

Similar to the exclusion of children with cultural differences documented in 1968, the exclusive practices of today continue to affect minority students at disproportionately higher rates. The Government Accountability Office (2020) issued a report summarizing data demonstrating this. In that report, the authors point out that, “while only 15.5% of public-school students were Black, about 39% of students suspended from school were Black” in the 2013–2014 school year (Government Accountability Office, 2020). Likewise, in 2019 they, “found that Black boys transferred to alternative schools at rates higher than any other group for disciplinary reasons, and that they, along with Hispanic boys and boys with disabilities, attended alternative schools in greater proportions than they did regular public schools” (Government Accountability Office, 2020). These disparities can be found among children as young as 3 and 4 years old.

This begs the question, if, as a society, we have the legal requirements to prevent school exclusion and the technology to provide access for all children, what is left to do to prevent children from being excluded from their education? The answer to this is simple: advocacy. As was written in the original report,

When public institutions, particularly one as basic as the public school system, fail to provide adequate services, it is an indictment of the total community. Failure in this indicates that private citizens, professional groups, and public officials either have not informed themselves about the exclusion of children from school, or they do not recognize it as an emergency requiring immediate action. In either case, it is clear that responsibility for this basic failure of the school system rests with the entire community. (Task Force on Children Out of School, 1970, p. 69)

The conclusion that the burden to mobilize to end school exclusion falls upon everyone still holds today. Fortunately, what also holds is that, “the most surprising aspect of this problem is that it need not exist. It is within our power to alter it” (Task Force on Children Out of School, p. 5). It will simply take collective action on two separate fronts. First, advocacy should occur on an individual level. These efforts should be focused on informing stakeholders of the rights of children so they can work to prevent exclusionary practices from occurring and stop them when they have. Second, sustained systemic advocacy should occur to remedy the underlying causes of exclusive practices, such as lack of funding, to provide the types of supports and programs these children need to access their education. Behavior analysts are well-positioned to be a part of both types of advocacy.

## Individual Advocacy

The first type of advocacy behavior analysts should engage in is individualized advocacy. This means educating stakeholders about the educational rights of children to prevent school exclusion. There are several resources that can assist in detailing educational rights including the procedural safeguards made available by each state education agency, modules developed by the IRIS Center (n.d.-a), and those resources made available via the Wrightslaw (n.d.) website. When discussing the topic of school exclusion, individual advocacy should center on informing stakeholders of the: (1) *Endrew F.* standard for educational progress; (2) protections afforded by manifestation determination reviews to prevent repeated suspension and informal removals; and (3) LRE mandate, which protects children from exclusion through restrictive placements such as modified day and homebound. These protections are described briefly below.

As stated previously, the *Endrew F. *v.* Douglas County School District *(2017) decision raised the standard for progress from, “anything more than *de minimis,*” which was applied in many regions of the country. To understand the implications, a brief overview of the case is warranted. Endrew F. was a child with autism enrolled in a public school. After years of not meeting his IEP goals, which were recycled from year to year, the family enrolled Endrew in a private, behavior-analytic school where he flourished. The family sought reimbursement for the tuition from the public school district, claiming the IEP was inappropriate. The 10th Circuit Court of Appeals initially rejected the family's claims, applying the “merely more than *de minimis*” standard that held that a school is only required to provide an IEP designed to provide anything more than a trivial or minor educational benefit (U.S. Department of Education, 2017). The Supreme Court disagreed, explaining that, “where grade level progress is not appropriate, educational programs must be appropriately ambitious in light of his circumstances” (*Endrew F.* v. *Douglas County School District*, p. 14). It is important to note the Supreme Court did not define precisely what level of progress is required. However, they were clear in stating that if a child was repeatedly not meeting their IEP goals, it was inappropriate and a FAPE was not provided. This Supreme Court ruling applies across all states, so it is now understood that all children should regularly meet appropriately challenging IEP goals. If a behavior analyst encounters a child who is not, they should feel comfortable informing the relevant stakeholders (e.g., parents, guardians, school representatives, other service providers) about the *Endrew F.* standard.

The second educational protection behavior analysts should be knowledgeable about to prevent school exclusion is manifestation determination reviews (MDR). The MDR says explicitly that if a child is removed from school for a total of 10 times in a school year for challenging behaviors, consecutively or cumulatively, the IEP team must convene and determine if the behaviors are a result, or *manifestation*, of the student’s disability (IDEA, 2004). Removal includes both formal in-school and out-of-school suspensions. It also includes informal removals, such as a principal calling a parent and asking them to be picked up early (OSEP Guidance, 2022). Except for a few exceptional circumstances (e.g., bringing a weapon to school), if the behaviors are a manifestation of the individual’s disability, the removals must end and the IEP team should take additional steps to ensure the child can access their school day (Wettach, 2017). These steps include the implementation of a functional behavior assessment and behavior intervention plan (IDEA, 2004). Thus, if a behavior analyst interacts with a child being repeatedly sent out of school, they should inform the relevant stakeholders of the MDR protections.

The third protection behavior analysts should be familiar with to assist in cases where children are facing school exclusion is the requirement that children be educated in the LRE that can confer progress. The IDEA (2004) outlines a clear continuum of alternative placements that each school district is responsible for providing. This includes the following, ordered from least to most restrictive: (1) general education classes; (2) separate classes; (3) separate schools, which can be either public or private and can provide services for just the school day or for the school day and residential services; (4) home instruction; and (5) instruction in hospitals and institutions. Children should be educated in the least restrictive setting that can confer educational progress (IRIS Center, n.d.-b). Therefore, if a child is physically present in a seemingly less restrictive environment on the continuum (e.g., general education class), but they are not learning and making progress in that setting, it may not be the least restrictive environment for them. The IEP team should evaluate what supports and services would allow for progress and find the setting that can provide those. It is important that settings such as homes and hospitals should be used in extremely rare cases, such as when a child has a medical condition that prevent them from safely attending school (IRIS Center, n.d.-b).

As described above, schools will sometimes place children on a modified day or homebound placement due to challenging behavior. However, this is not consistent with the least restrictive environment provision of IDEA (2004). As explained in the Duke University Law School’s *Parent’s Guide to Special Education*:

Neither a “modified day” nor a homebound placement is appropriate as a response to a child’s behavior problems. A child with a disability who has difficulty following school rules requires special education to address the behavioral issues. Reducing the number of hours at school because it reduces the child’s opportunity to misbehave is not consistent with providing the child a free, appropriate public education, does not help the child learn to behave more appropriately, and discriminates against children with behavior problems by limiting their education. (Wettach, 2017, pp. 100–101)

Therefore, if the child has challenging behaviors that prevent them from accessing an educational setting, the school should increase the supports and services provided to ensure the child can access that setting. If the supports and services are not feasible in that setting, they should look for a setting that can provide the needed supports and services. If the school district does not have the necessary supports and services, they should explore options to obtain them such as contracting with outside providers to supply those services (e.g., conducting an FBA) or placement at private specialty schools, as was the case with Endrew F.

A final note on individualized advocacy is that behavior analysts should always engage in these activities while being mindful of their ethical obligations. The *Ethics Code for Behavior Analysts* repeatedly instructs behavior analysts to act in the best interest of clients (Behavior Analyst Certification Board [BACB], 2020). In the context of this discussion, that means informing relevant stakeholders of the child’s educational rights to prevent school exclusion and always advocating for what is in the best interest of the client (BACB, 2020). However, the same ethics code calls upon behavior analysts to avoid multiple relationships and practice within their scope of competence (BACB, 2020). There may be times when behavior analysts have these conversations and feel they are being asked to fulfill multiple relationships or give advice outside their competence. For example, there will be times when the IEP team cannot agree on a course of action. The IDEA (2004) affords several processes to navigate these disputes such as facilitation, mediation, filing state complaints, and due process. These can be incredibly complicated situations to navigate, which may lead to a caregiver asking a behavior analyst to act as an educational advocate. Although behavior analysts may have the expertise to work as in this role, if they choose to do so they must be careful to have clear guidelines regarding their role and not engage in multiple relationships (e.g., serving as both the child’s behavior analyst and educational advocate). If this clarity is not possible or if an analyst does not feel they have the expertise to serve as an advocate, they should refer to other professionals in accordance with the referrals section of the ethics code (BACB, 2020). For example, each state has a center to support caregivers with navigating the special education process and a protection and advocacy agency for individuals with disabilities. Relevant centers can be found through the Center for Parent Information and Resources’ Find Your Parent Center tool (Center for Parent Information & Resources, n.d.) and the member agencies of the National Disability Rights Network (n.d.). Finally, if the situation is particularly complex and unclear, analysts can take actions such as seeking advice from colleagues and/or mentors, search relevant literature for guidance, and use guidance on ethical decisions (BACB, 2020).

Engaging in individual advocacy and encouraging stakeholders to pursue their existing educational rights will increase the fidelity with which important protections are implemented. Stakeholders need to fight for these rights to ensure all students access their education. Behavior analysts can assist in this by informing them of the *Endrew F.* standard of progress, MDR protections, and LRE requirements. As described above, more information on those topics can be found through resources such as the procedural safeguards notice distributed by state education agencies, the modules developed by the Iris Center (n.d.-a), and resources from Wrightslaw (n.d.).

## Systems Advocacy

Although engaging in individual advocacy is critical to ensure specific children are no longer excluded from their education, it will not fix the underlying issues that have led to exclusive school practices in the first place. For example, the references above clearly describe that children often encounter school exclusion due to challenging behaviors (AP News, 2021; Disability Rights North Carolina & North Carolina Department of Public Instructions, 2017; Kolodner & Ma, 2022; Stites, 2022). These children may have extremely complex needs that require sophisticated services and programs to support. As stated before, the technology and models to serve even the most complex children exist, thanks in large part to the field of behavior analysis. However, school districts working with a fixed budget may not have the funding to invest in or create the sophisticated programs these children need to access their education. As such, these services and programs may simply not exist in some areas. This is why systems advocacy on this issue is critical for behavior analysts to engage in. By doing so, the underlying issues leading to school exclusion can be addressed and systems can be developed and funded to ensure all children can access their education.

Behavior analysts are well positioned to engage in systemic advocacy, as we know that in order to change the behavior of individual decision makers, the environmental contingencies must change to enable those in power to have access to a better array of choices for our students. The field simply needs direction on how to focus advocacy efforts on the issue of school exclusion. Fortunately, the original Task Force on Children Out of School (1970) provides a roadmap to accomplish just that. In the Task Force’s original document, the authors identify several entities needing to implement action to address the school exclusion issue. These included education departments, health and human services departments, school boards, legislatures, and public and private service providers (Task Force on Children Out of School, 1970, p. 69).

These same entities need to be engaged today to address this issue, if in slightly different ways. Today, education departments, school boards, and legislatures should be made aware of the issue, informed of the broader racial impacts of these practices, and educated on and encouraged to fund systems that can educate these children. In addition, organizations who support, but are not directly responsible for the education of, these children should be engaged to assist in systemic advocacy efforts. These organizations include health and human services departments, behavioral health service providers, and professional trade organizations that support these children (e.g., the Council for Autism Service Providers, Association for Professional Behavior Analysts, Autism Speaks).

### Education Departments, School Boards, and Legislatures

Education departments, school boards, and legislatures have direct control and responsibility on this issue. They are responsible for the implementation of the law and the provision of effective services. As such, behavior analysts should work to engage these entities to educate them on the issue, describe the broader racial impacts, and encourage them to develop models that can ensure all children access their education. Regarding educating them on the issue, the law protecting these children exists, as described above. However, many of the individuals serving in these entities do not know how pervasive school exclusion continues to be. Therefore, it is incumbent upon those who support the affected children to make policymakers aware of the extent of this issue and urge them to increase the enforcement of existing protections so that children stop being excluded. This can be accomplished with actions as simple as reporting stories of and data on school exclusion to these bodies. It can also be as complex as coordinating events such as trips to state capitols. Whatever the action, simply alerting policymakers to the fact that children are consistently excluded from school can lay the groundwork towards systemic changes to address the issue.

In addition to the core issue of school exclusion, education departments, school boards, and legislatures should be educated on the racial disparities that occur with these practices. As behavior analysts, we have an ethical duty to help elevate access to all by pointing out access, equity, and school exclusion issues. For instance, the death of George Floyd and other Black men has brought long-term racial disparities and system inequality to the forefront. Behavior analysts can work with other stakeholders to evaluate and highlight where these racial disparities are occurring. One of the most basic ways they can do this is through data. Behavior analysts know how to collect data, and they should use that knowledge to both ask schools to record their demographic data of school exclusions and work to set goals for the successful return of students to the academic environment.

Last, is the need to engage these groups on developing programs and services that can successfully educate all children. As stated previously, the children being excluded are generally those in the greatest need of additional supports and services because they are the most complex. There may not be any programs that can properly support them in a region. Simply bringing these children back into existing school programs will not ensure they will access educational progress. School systems need additional resources, training, and funding to implement the programs necessary for these children to access their education. Fortunately, there are models for how to do this, such as the network of Chapter 766 schools that were developed in Massachusetts to include even the most complex children, many of which are behavior analytic. Policymakers need to know about these models so they can invest in developing them. Behavior analysts can mobilize to advocate for funding and development of these programs so the education system can better serve students with complex needs. Then, as investment in these programs occurs, behavior analysts will have the resources needed to expand our technology to underserved areas.

### Additional Stakeholders

In addition to advocating to those with direct responsibility for children’s access to education, systems advocacy should include coordination across other relevant stakeholders. Policymakers are generally responsible for many more areas of society than their personal expertise covers. Even within education departments, few will be focused on special education issues. Thus, policymakers rely on the knowledge of others to drive their decisions. If multiple stakeholders give contradicting suggestions, it is easy to dismiss those suggestions. This means we must coordinate messages and requests across organizations and disciplines to ensure everyone supporting these children speak with a singular message. Given the central importance of school to children, that message is that, “any child out of school or in need of special services represents an emergency situation” (Task Force on Children Out of School, 1970 p. 76), and models of service should be developed to include all children in school.

The first type of stakeholder to coordinate messaging with is entities that are often called on to support children who are excluded from school. This includes organizations such as health and human services departments, behavioral health service providers, and insurance funded therapies. If these groups do not view school exclusion as the emergent need it is, their message to policy makers will reflect other priorities. For instance, they may advocate for more funding for their organization because they are bearing an increased burden by supporting children who are not in school for a full day. However, this would be shortsighted, as it does not address the underlying issue of children not accessing their education. A more appropriate message for these groups would be to follow the recommendations described above. In particular, to raise awareness on the issue, explain the racial inequities, and advocate for additional funding for programs that ensure children can remain in school for a full day and make educational progress. Behavior analysts who interact with or work for these organizations should work to ensure they support that message.

The second group to coordinate with and encourage advocacy on this issue is professional trade organizations (e.g., the Council for Autism Service Providers, Association for Professional Behavior Analysts). Some of these organizations are already familiar to behavior analysts and have worked in the field for some time. This also includes organizations that have been less directly involved with advocacy for behavior analysis, but should be engaged on this issue as they support children. This would involve groups like organizations that support people with disabilities (e.g., state protection and advocacy organizations, self-advocates), groups focused on educational issues, and groups focused on supporting children. It also means enjoining representatives of the very entities we are advocating towards to join in the mission, such as representatives from the education system. The coalition should be as broad as possible, for if all the various groups supporting these children are approaching policymakers with the same message, they will be more inclined to act to remedy the problem. This should be attainable with such a simple mission of finally ending the practice of excluding children from school.

Systemic advocacy is essential to addressing the underlying issues that have led to school exclusion. This is because, despite the best of intentions, many schools simply lack the capacity to ensure children with more complex needs can access their education. Behavior analysts can engage in systems advocacy in practical ways. First, they can engage those with direct responsibility for ensuring educational access by raising the issue of school exclusion to their attention, detailing the racial disparities that occur, and educating them on and advocating for developing models that can successfully educate these children. Second, they can engage with other stakeholders, such as service providers and professional organizations, to ensure everyone is communicating with a consistent message on this issue. If all parties are regularly communicating with education departments, school boards, and legislatures with a consistent message, there is a much greater likelihood the underlying issues will be addressed and more resources will be deployed to develop effective programs for these children.

## A Final Note on Framing the Issue

Behavior analysts must be careful in framing this issue. The field of behavior analysis has a well-developed technology to facilitate successful access to education for even the most complex children. However, we must remind ourselves that, in contrast to recent advocacy efforts, access to behavior analysis alone is not the goal. The goal is access to education for all children because that is what the federal entitlement is. Without question, behavior analysis will play an essential role in facilitating that. This is an important and significant advantage to our role in advocacy because when policymakers question how it is possible to educate these most complex of children, we can confidently point to the successes of our field in doing just that. However, we must remember that in this case, behavior analysis is a means to an end. Behavior analysis may be the method that allows a child to access their education. However, it is access to educational progress we must advocate for, not merely the service of a behavior analyst. As an example, a family may be counseled to withdraw their child from school to enroll full-time at an ABA clinic operating under a medical model. However, even though the child in this scenario would have access to ABA, this would still represent a serious violation of the child’s rights because they are forced to choose between receiving the educational services they are entitled to and the medical services they qualify for. They should not have to choose. They should have access to a system that affords both where they receive a robust educational program where they progress with all relevant skills (e.g., academic, functional, adaptive, communication) and medically necessary ABA to support improved skills in the home and community.

This frame is also applicable when engaging in individual advocacy to ensure access to education. When working on activities that may directly affect educational services and/or programming, we must understand the special education laws better and ensure that our work enhances the likelihood of children fully accessing their education. This is true regardless of whether the funding comes from the school district directly (e.g., as an employee or contractor) or from another source where the work will be used to drive educational decisions (e.g., independent educational evaluation, collaboration). For instance, when writing assessments that may be used to determine educational services and/or programming (e.g., a functional behavior assessment), we must directly link the analysis and recommended interventions to children’s educational access. Identifying the variables controlling a challenging behavior and designing an evidence-based intervention is not enough. Instead, the analyst should clarify how the targeted behaviors prevent the child from accessing their education and how the interventions recommended will remedy that. Without this explicit connection, schools often separate a student’s challenging behavior from educational access.

Therefore, even in our daily work, we must remember that when interfacing with schools, behavior analysis is the tool for achieving the goal of ensuring children access their legal right to a FAPE.

## Conclusion

Over 50 years have passed since the original report documenting the crisis occurring in Boston Public Schools, which was the exclusion of children from school. Many strides have been made to rectify the crisis. Laws and regulations have been passed, progressively affording more protections to children to ensure they access their education. In addition, the science of behavior analysis has developed an increasingly effective technology to support these very complex children so they can access their education. Despite these advances, the original crisis of exclusion from school persists for too many children. It continues to be a crisis that requires coordinated and sustained community advocacy to solve.

Behavior analysts are uniquely positioned and skilled to advocate on this issue. We can affect it on an individual level because we serve many of the children affected by this issue. Therefore, we can effectively educate stakeholders of the educational protections all children are entitled to including the *Endrew F.* standard, MDRs, and LRE requirements. We can also address it on a systemic level, as we have done successfully on other issues. We can do this by elevating the issue to education departments, school boards, and legislatures. We can explain the racial disparities that exist, and point to models that are worth developing because they effectively include even the most complex children in education. We can also work across stakeholders to ensure consistent messaging, increasing the likelihood of developing such programs.

In closing, we could make a financial argument, pointing out that states would save money over time by investing early in the highly sophisticated education these most vulnerable children need to make progress. However, that argument fails to meet the moment. Instead, we will continue to follow the lead of the authors of that original report by looking at this in human terms. In particular, we close by reminding everyone that we are called to advocacy on this issue because, “we carry the responsibility for the very development of children” (Task Force on Children Out of School, 1970, p. 71), for without us, we leave another generation of children behind.

## Data Availability

Data sharing is not applicable to this article because no datasets were generated or analyzed during the current study.
